# Development and Validation of HPLC Method for the Determination of Pregabalin in Capsules

**DOI:** 10.4103/0250-474X.73935

**Published:** 2010

**Authors:** G. B. Kasawar, M. N. Farooqui

**Affiliations:** Dr. Rafiq Zakaria Campus, Post Graduate Studies and Research Centre, Moulana Azad College Rouza Bagh, Aurangabad-431 00, India

**Keywords:** Method development, pregabalin, RP-HPLC, validation

## Abstract

A simple, precise, specific, and accurate reverse phase HPLC method has been developed for the determination of pregabalin in capsule dosage form. The chromatography was set on Hypersil BDS, C8, 150×4.6 mm, 5 μm column using photodiode array detector. The mobile phase consisting of phosphate buffer pH 6.9 and acetonitrile in the ratio of 95:05 with flow rate of 1 ml/min. The method was validated according to ICH guidelines with respect to specificity, linearity, accuracy, precision and robustness. Lower limit of quantification is 0.6 mg/l. The pregabalin sample solution was found to be stable at room temperature for about 26 h.

Pregabalin, an antiepileptic drug similar to gabapentin produces its actions by binding to the alpha2-delta (α2δ) subunit of the voltage-gated calcium channels[1]. The influence of fluctuating temperature and humidity conditions that might occur during transportation of drug products can be estimated using stability analysis of a drug[2]. Few reports on determining pregabalin content in pharmaceuticals have been published, involving spectrophotometric, spectrofluorimetric methods[3] and precolumn derivatization method using internal standard[4,5]. Synthesis and characterization of pregabalin lactose degradation product was reported[6]. Determination of pregabalin without pre-column derivatisation using RP-HPLC has not been reported thus far. Therefore, in the present investigation an attempt has been made to determine pregabalin in solid dosages form using RP-HPLC without pre-column derivatization of analyte and without internal standard. The assay is calibrated over the range of 500 μg/ml to 1500 μg/ml and without derivatization of analyte also the proposed method can quantify (LOQ) at least 0.61 μg/ml and can detect (LOD) at least 0.23 μg/ml. The developed method has been validated showing the method accuracy, linearity and reproducibility. Validation procedure was mainly based on the ICH guideline[7].

Pregabalin working standard and pregabalin drug product (Lyrica) were procured from the market; Potassium dihydrogen orthophosphate, potassium hydroxide and HPLC grade acetonitrile used were obtained from Merck Ltd, Mumbai, India. Water used for the study from Milli-Q system (Millipore, Bedford, USA).

Waters 2690 series LC system with photodiode array detector with inbuilt auto injector (Waters, Alliance) was used for method development and validation. Data acquisition and system suitability calculations were carried out using Waters Empower software. Hypersil BDS C8, (150 × 4.6) mm, 5 μ, (Thermo Fisher Scientific, Waltham MA, USA) column using isocratic mobile phase consisting of a mixture of potassium phosphate buffer (adjusted to pH 6.9) and acetonitrile in the ratio of 95:05 (v/v) was used throughout the analysis. The flow rate of the mobile phase was 1 ml/min. Detector signal was monitored at a wavelength of 200 nm. The column oven was maintained at 30° and injection volume was 20 μl.

Accurately weighed sample of pregabalin (50 mg) working standard was transferred to a 50 ml volumetric flask and 30 ml of mobile phase was added and sonicated to dissolve the sample completely. The solution was made up to the mark and filtered through 0.45 μ nylon membrane filter. Capsule powder equivalent to 100 mg of pregabalin (about 454 mg of pregabalin capsule powder of 25 mg strength) was accurately weighed and transferred in to a 100 ml volumetric flask; 60 ml of mobile phase was added and sonicated with occasional shaking for 30 min. The solution was cooled to room temperature and diluted to volume with the diluent. The final solution was filtered through a 0.45 μ nylon membrane filter.

In order to develop a suitable and robust LC method for the determination of pregabalin, various chromatographic conditions were employed using different stationary phases like C8 and C18 with different column dimensions and different mobile phase containing buffers like phosphate and acetate with different pH ranging from 6.5 to 7.5 and using organic modifiers like acetonitrile and methanol in the mobile phase. Finally, the mobile phase consisting of aqueous 1.2 g monobasic potassium phosphate per liter with a pH 6.9 and acetonitrile in the ratio of 95:05 (v/v) at a flow rate of 1.0 ml/min using Hypersil BDS C8, 150×4.6 mm,5 μ column was found to be appropriate. The column oven was maintained at 30°. The compound has no chromophore (fig. 1), as a result the compound would give no characteristic spectra. Hence, 200 nm wavelength has been chosen for the detection in the analysis for better sensitivity. Under these optimized conditions, the analyte peak is very symmetrical. The typical HPLC chromatogram was scanned at 200 nm (fig. 2). The developed method was validated according to the ICH guidelines with respect to specificity, linearity, accuracy, precision and robustness.

**Fig. 1:**
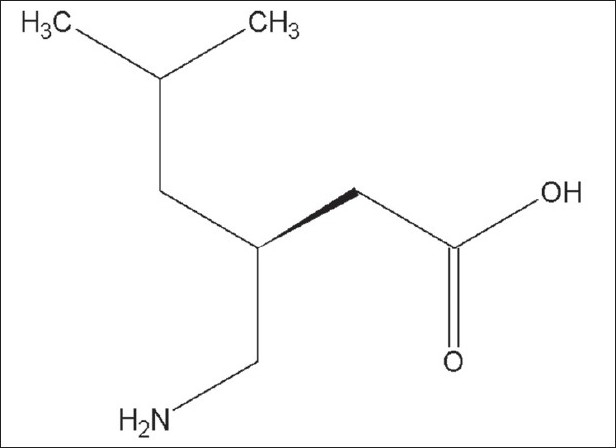
Chemical structure of pregabalin

**Fig. 2:**
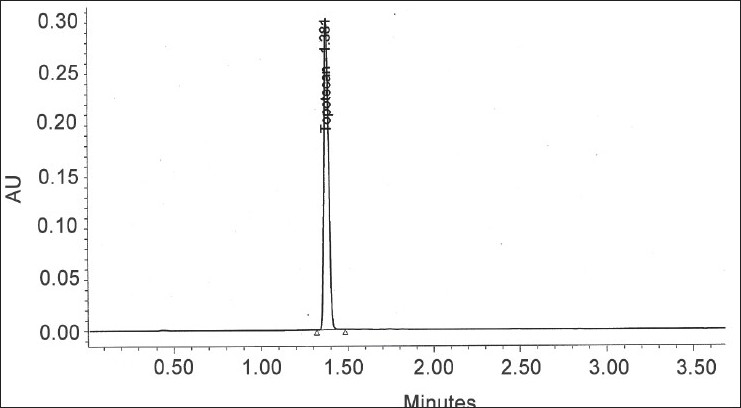
Typical HPLC chromatogram of sample solution

The specificity of the method was checked by injecting blank solution, excepient solution, sample solution and sample solution spiked with all related known impurities at 1% level in triplicate. There was no interference from blank and excepients at the retention time of analyte peak. The % difference between the assay of spiked sample results and sample solution results was found 0.58% which is less than 1%. Specificity was also checked by exposing the sample under stressed conditions like heat (105°), light (1.2 million lux), humidity (40°/75% RH), acid (5N HCl), base (5N NaOH) and peroxide (30% w/v H_2_O_2_). Percent degradation achieved was about 22% in acid-treated sample, 24% in base-treated sample, 19% in peroxide-treated sample and 2 to 5% degradation was observed in sample exposed to thermal, light and humidity. The peak purity was checked by confirming homogeneous spectral data for pregabalin in each stressed conditions and it indicates that there is no interference from related impurities and excepients.

Pregabalin showed linearity in the concentration range of 0.5 mg/ml to 1.5 mg/ml. The linear regression equation and correlation coefficient (R^2^) is y=1353x-1763 and 0.99982, respectively (Table 1). The accuracy of the method was determined by adding known amount of drug substance corresponding to three concentration levels of 50, 100 and 150% (i.e. 50, 100 and 150 mg) of target analyte concentration along with the excipients in triplicate. The accuracy was expressed as the percentage of analyte recovered by the assay method. It was confirmed from results that the method is highly accurate (Table 2).

**TABLE 1:** REGRESSION STATISTICS

Statistics	Results
R square	0.99982
Slope	1352.87
Intercept	1763.32
t Stat	489.564
*P*-value	3.39E-19
Lower 95% of slope (confidence interval)	1344.91
Upper 95% of slope (confidence interval)	1357.64

**TABLE 2:** ACCURACY DATA OF PREGABALIN

Recovery level (%)	Amount added (mg)	Amount recovered (mg)	Recovery (%)	RSD (%)
50	49.88	50.26	100.72	0.03
100	100.20	100.59	100.39	0.60
150	150.09	150.95	100.57	0.20

Repeatability of the method was checked by carrying out six independent assays of test samples against qualified working standard. Intermediate precision was performed by analyzing the samples by two different analysts using different instrument and different columns on different days. The percentage relative standard deviation of assay values for repeatability (n=6) is 1.67% and for intermediate precession (n=12) is 1.66%.

Robustness of the method was performed by deliberately changing the chromatographic conditions. The flow rate of the mobile phase was changed from 1.0 to 0.8 and 1.2 ml/min. The organic strength of mobile phase was varied by ±10% while pH of buffer was varied by±0.2 units. The standard solution and three different sample preparations were injected in each varied conditions and checked the assay. In all deliberately varied conditions, the percentage relative standard deviation for the assay values (n=3) for pregabalin were found to be well within the acceptance limit of 2%. The tailing factor for the pregabalin peak was found to be < 1.5 indicating the method robustness.

Proposed HPLC method is specific, accurate and precise for the determination of pregabalin from its pharmaceutical dosages form. The described HPLC method is suitable for routine analysis and quality control of pharmaceutical preparation containing pregabaline active pharmaceutical ingredient.
